# Multispectral fingerprinting for improved in vivo cell dynamics analysis

**DOI:** 10.1186/1471-213X-10-101

**Published:** 2010-09-24

**Authors:** Paul M Kulesa, Jessica M Teddy, Miranda Smith, Richard Alexander, Cameron HJ Cooper, Rusty Lansford, Rebecca McLennan

**Affiliations:** 1 Imaging/Kulesa Lab, The Stowers Institute for Medical Research, (50th St) Kansas City, (64110), USA; 2 Department of Anatomy and Cell Biology, The University of Kansas School of Medicine, (Rainbow Blvd), Kansas City, (66160), USA; 3 Division of Biology, California Institute of Technology, (E. California Blvd), Pasadena, (91125), USA

## Abstract

**Background:**

Tracing cell dynamics in the embryo becomes tremendously difficult when cell trajectories cross in space and time and tissue density obscure individual cell borders. Here, we used the chick neural crest (NC) as a model to test multicolor cell labeling and multispectral confocal imaging strategies to overcome these roadblocks.

**Results:**

We found that multicolor nuclear cell labeling and multispectral imaging led to improved resolution of in vivo NC cell identification by providing a unique spectral identity for each cell. NC cell spectral identity allowed for more accurate cell tracking and was consistent during short term time-lapse imaging sessions. Computer model simulations predicted significantly better object counting for increasing cell densities in 3-color compared to 1-color nuclear cell labeling. To better resolve cell contacts, we show that a combination of 2-color membrane and 1-color nuclear cell labeling dramatically improved the semi-automated analysis of NC cell interactions, yet preserved the ability to track cell movements. We also found channel versus lambda scanning of multicolor labeled embryos significantly reduced the time and effort of image acquisition and analysis of large 3D volume data sets.

**Conclusions:**

Our results reveal that multicolor cell labeling and multispectral imaging provide a cellular fingerprint that may uniquely determine a cell's position within the embryo. Together, these methods offer a spectral toolbox to resolve in vivo cell dynamics in unprecedented detail.

## Background

The vertebrate embryo performs a wonderful orchestration of cell movements to distribute cells into precise locations in a programmed, stereotypical manner. One example of this is how the embryo sculpts the migratory pattern of a highly invasive, multipotent population of cells called the neural crest (NC). NC cells form all along the dorsal neural tube, but are shaped into discrete migratory streams and differentiate into diverse cell types that include neurons, glia, pigment, bone and cartilage [1,2]. Although cell tracing studies have shed light on the migratory pathways and developmental potential of the NC, the dynamics of single NC cell movements and cell contact have remained unclear.

Key to our understanding of the NC cell migratory pattern is the need to observe how single cell behaviors lead to a coordinated, directed migratory stream. However, traditional single color cell labeling approaches (such as microinjected fluorescent dextrans or lipophilic dyes (for example, DiI), tissue chimeras, or membrane green fluorescent protein (GFP)) fail to uniquely identify a single NC cell and its contacts with neighbors in dense migratory streams. This is due in part to the inability to uniquely identify a cell after two or more cell trajectories cross in space and time, and when cellular processes and cell borders overlap. In addition, the resolution of an optical microscope to determine the smallest distance between two points during live embryo imaging is limited by a number of factors. These include cell labeling efficiency, tissue properties that affect the light path, and the long working distance between the embryo and objective. Thus, a major goal of NC dynamics analyses, shared by many developmental systems, is the need for better cell labeling and imaging tools that more accurately track single cells and cell dynamics in the embryo.

The use of multicolor fluorescent proteins [3] and targeted cell labeling have emerged as powerful tools with the potential to elucidate the cellular choreography of complex morphogenetic events. Multicolor cell labeling schemes tested mostly on cultured cells have benefitted from spectral imaging and linear unmixing. Spectral detection has evolved from using a liquid crystal tunable filter to collect the emission spectrum of each pixel within an image, to more sensitive and flexible detector technologies that yield spectral resolutions down to ~1 nm. Mathematical analysis then determines the contribution of different fluorophores to the total signal, using reference spectra. Spectral imaging has been validated on multicolor labeled cells in culture [4,5], providing an excellent framework to extend the techniques to live tissue and embryos with both 3D multispectral confocal and 2-photon laser scanning microscopy.

Within intact embryos, several reports have described successful multicolor cell labeling approaches to visualize tissue architecture and cell cytoskeletal elements, in zebrafish [6], *C. elegans *[7], *Drosophila *[8], mouse [9,10] and chick [11]. An exciting approach to better identify single cells within the embryo and build a dynamic fate map uses a 1-color nuclear targeted (H2B-FP) fluorescent reporter strategy [12-14]. However, although a nuclear localized fluorescence signal offers some clarity to single cell identification in sparse tissues, it is still very challenging to resolve single cells, their precise trajectories, and cell contact dynamics in dense cell migratory streams and tissues within the embryo.

Here, we addressed two simple questions. First, how can we more accurately identify and trace single cells in the developing embryo. Second, can we better resolve cell contact dynamics when cell protrusions or borders overlap between neighboring cells. We used the chick NC as our model system, since we have previously developed an in vivo platform to test emerging cell labeling and imaging strategies [11,15,16]. To address the first question, we compared the accuracy of identifying individual fluorescently labeled cells in 3D tissue sections from chick embryos (hindbrain level) labeled with either 1-color (membrane (DiI), membrane (Gap43-GFP), or nuclear (H2B-RFP)), or 3-color (H2B-YFP, -RFP, and -GFP or -CFP) fluorescent reporters. We developed and simulated a computer model that distributed and counted a varying number of cells within a fixed rectangular region to predict the accuracy of object counting in multicolor labeled tissues with increasing cell densities. To test how a 3-color nuclear targeted cell labeling strategy might improve cell tracking, we measured the spectral identity of NC cell neighbors prior to and immediately after cell trajectory crossings in 4D (3D+time) multispectral confocal imaging sessions of 3-color H2B-FP labeled chick embryos. To address the problem of resolving cell contact dynamics, we tested 3-color combinations of cell membrane (Gap43-FPs) and nuclear (H2B-mCherry) fluorescent plasmids and analyzed overlapping cell protrusions in neighboring cells. Lastly, we compared channel versus lambda scanning modes and measured the time to collect and manipulate 3D multispectral confocal z-stacks of 3-color labeled embryos.

## Results

### A comparison of single cell identification in vivo using 1-color and 3-color cell labeling and 3D multispectral imaging

**Figure 1:**
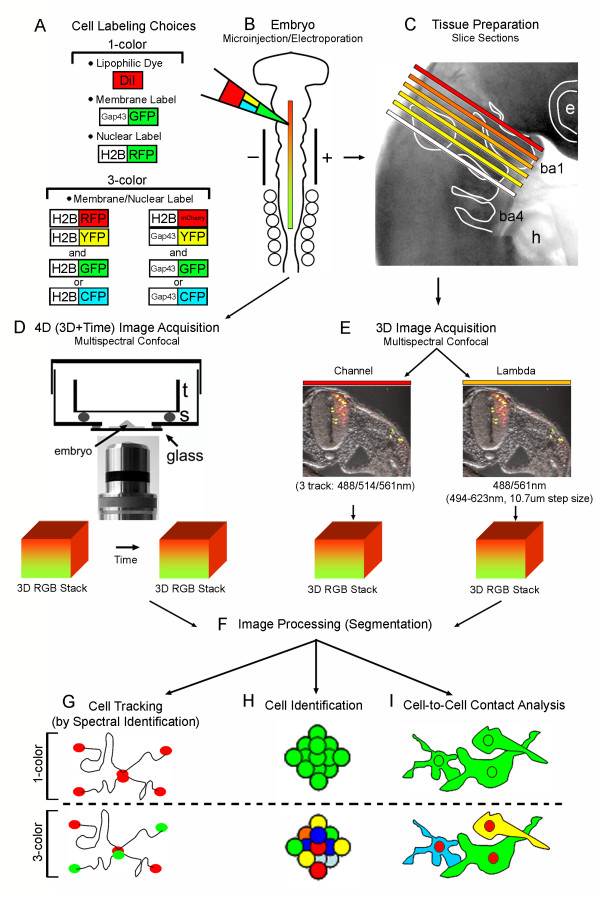
**Schematic Overview of Multicolor Cell Labeling and Multispectral Imaging Approach**. (A) Four different cell labeling strategies were compared in chick embryos, including lipophilic dye (DiI), membrane label (Gap43-GFP), 1-color nuclear label (H2B-RFP) or 3-color (H2B-YFP, -RFP, -CFP or GFP), and membrane/nuclear labels (Gap43-YFP, Gap43-CFP or Gap43-GFP, and H2B-mCherry). (B) For each of the different experiments, one of the four different labeling options were microinjected into the lumen of the chick neural tube and the embryo was electroporated. (C or D) After re-incubation, the embryos were either sectioned in the hindbrain region and mounted on a glass slide with a coverslip or mounted as whole embryos for live imaging. (D-E) Each slice was imaged using either channel or lambda scanning in confocal multispectral imaging to obtain a 3D or 4D data set. (F) Images were processed to ensure that background was reduced for accurate cell identification in spot detection. (G) NC cells were tracked throughout time with spectral identification using a combination of AIM line intensity profile and Imaris spot detection. (H) Alternatively, the 3D static data were analyzed in Imaris with spot detection for automated cell identification and manual cell count. (I) Filament tracing (Imaris) was used to measure cellular processes on individual cells and provide a picture of cell-cell interactions.

To determine the most accurate method for single NC cell identification and tracking, we performed a comparison of labeling techniques and manual versus automated cell identification (Fig. 1). To label premigratory NC cells, chick embryos (HH St 9-10) were injected and/or electroporated with four different fluorescent cell labeling strategies. Specifically, we compared DiI (lipophilic dye), membrane GFP (Gap43-GFP), 1-color nuclear targeted (H2B-RFP) and 3-color nuclear targeted (H2B-YFP, -GFP or -CFP, and -RFP) labeling strategies. Embryos were harvested 24 hours after injection and re-incubation, and tissue sections (at the level of rhombomere 4 in the head) were prepared from fixed embryos for 3D multispectral confocal imaging (Fig. 1). Cells within the dorsal neural tube (including premigratory NC cells) and migratory NC cells were identified and counted from 2D maximum intensity projections by visual inspection and in an automated manner using commercial software. Data were analyzed to determine the cell labeling strategy that provided the most accurate cell identification and tracking. In addition, we tested the ability of a multicolor cell labeling approach to more accurately detect single NC cell movements and cell contact dynamics, using 3D and 4D multispectral confocal and 2-photon imaging (Fig. 1).

### DiI and membrane GFP cell labeling led to an over- and under-estimation of cell number

**Figure 2:**
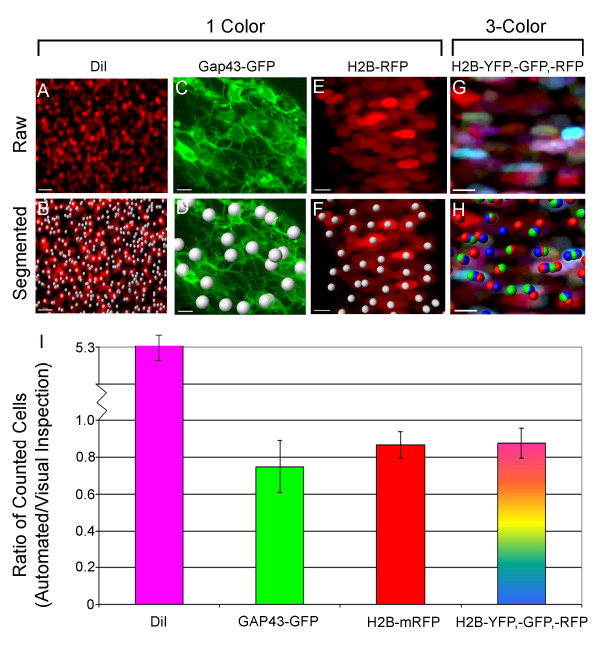
**A Comparison of Cell Identification in 1-Color and 3-Color Labeled Chick Embryo Tissues**. (A-H) Four different groups of embryos HH St 9 (n = 5-6 in each set) were injected with 1-color (DiI, Gap43-GFP, or H2B-RFP) and 3-color (H2B-YFP, RFP, CFP or GFP) combinations. (A, C, E, G) After reincubation of 18-24 hours, the embryos were sectioned and typical confocal lambda and channel z-stacks were obtained. (B, D, F, H) The 3D reconstructions of each labeling method are shown with the corresponding segmented spot detection. (I) The graph highlights the differences between the cell identification for the different labeling strategies. The error bars show +/- one standard deviation. The scale bars are all 10 um.

To compare the four cell labeling strategies, we first analyzed differences in NC cell identification using visual inspection and automated cell counting. We anticipated that when NC cells were sparsely distributed, there would not be significant differences in cell identification since most segmentation software can detect sparsely distributed cells. Thus, we analyzed embryos in which the average NC cell density was approximately 2-3 cells (each cell nucleus is ~10 um in diameter) per 100 squared microns (10 um × 10 um) subregions, in transverse sections. To compare NC cell identification, we measured the number of cells counted automatically, divided by the number of cells counted by visual inspection. Our initial speculation that DiI was not sufficient for accurate cell counting in dense cell populations proved to be correct (Fig. 2). DiI was significantly less accurate in NC cell identification (Fig. 2). Cell counting software estimated over 5 times as many cells as were identified by visual inspection (Fig. 2). This was due in part to the nature of lipophilic dye labeling, since DiI-labeled cells often had particulates of dye in the cell membrane leading to the false impression of multiple single cells. In contrast, automated cell identification of Gap43-GFP labeled embryos led to an under-estimation of cell number compared to visual inspection (Fig. 2). This was in part due to difficulties to accurately resolve neighboring cell borders (Fig. 2).

### Nuclear cell labeling improved NC cell identification in densely populated migratory streams and 3-color nuclear labeling decreased the time and effort to identify single cells

To determine whether there was a significant difference in NC cell identification in multicolor labeled embryos, we compared automated versus visually inspected 3-color and 1-color H2B-FP labeled embryos. We found that automated detection of 1-color H2B-RFP labeled NC cells identified approximately 87% of the total number of cells that were counted by visual inspection (Fig. 2). Surprisingly, we found that the addition of more colors (3-color H2B-FPs) only showed a very slight increase to improve the difference between automated and visually inspected NC cell identification (88% compared to 87% (Fig. 2, columns 3 and 4). However, we did find that 3-color H2B-labeled cells within dense tissue samples were more distinct from neighboring cells and thus made it faster to visually distinguish and count NC cells. Differences in fluorescence intensity between cells, and neighboring cells with the same fluorescent reporters, still made it difficult to resolve single cells. Thus, for NC cell identification, the most efficient (speed and accuracy) approximation of cell number was achieved in 3-color H2B-FP labeled embryos at ~90% of the total cell number and the worst case was when cells were labeled with DiI.

### Computer modeling predicted that 3-color nuclear labeling would improve automated object counting accuracy for increasing tissue density

**Figure 3:**
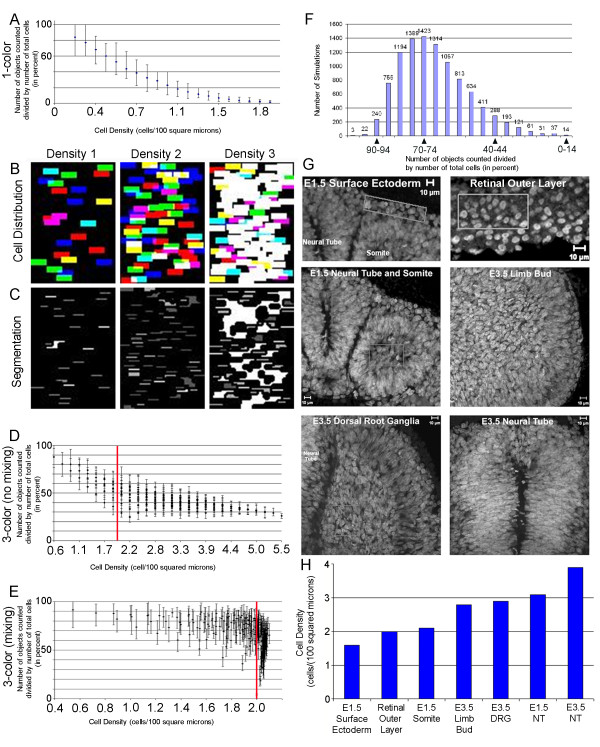
**Computer Model Simulations Predict Improved Counting Accuracy for Increasing Cell Densities in 3-Color versus 1-Color Cell Labeling Strategies**. (A) For a 1-color nuclear labeling approach, computer model simulations predict the number of objects counted versus cell density. (B-C) Each initial condition for the simulations consisted of seeding a rectangle (approximating a dense tissue within the embryo) with a number of cells (represented by smaller rectangles) and running each simulated image through a custom segmentation routine (Additional File 1), then calculating the number of objects. (D) For 3-color (non-mixing) and (E) 3-color (mixing) cell labeling strategies, the computer predictions are shown for number of objects counted versus cell density. The red line marks the position of cell density = 2.0. (F) The bar graph histogram shows the number of objects counted versus the count of simulations at all the different densities in the 3-color (mixing) cell labeling strategy. The percentage is binned into five percent increments. (G) Individual images of DAPI stained embryos shows the different densities within the developing chick embryo. (H) The different regions represent E1.5-E3.5 (E = embryonic day) chick embryos, with surface ectoderm, neural tube, somite, dorsal root ganglia, retinal outer layer, and limb bud densities represented in the graph. Each tissue density can be correlated with the cell densities simulated in the computer model for (A) 1-color, (D-E) and 3-color cell labeling strategies.

We constructed a computer model to simulate automated counting of NC cells (Fig. 3; Additional File 1). Specifically, we compared the predicted number of objects that could theoretically be detected by an algorithm as a function of increasing tissue density. We created three different simulation scenarios using 1-color, 3-color (no mixing; an individual cell can only have one color), and 3-color (mixing; an individual cell can have more than one color and up to three). The computer simulations were completed in three different channels (RGB) to simulate the different colors associated with 3-color nuclear cell labeling. For each of these 3 cell labeling scenarios, we averaged the results from 10,000 simulations and calculated a graph for each scenario (Fig. 3).

In the simulations of 1-color nuclear cell labeling, as the density increased there was a sharp decrease in the number of counted objects (Fig 3A). At a cell density of ~0.6 cells/100 squared microns, there were less than 50% of objects counted (Fig 3A) and at a density of ~1.6 cells/100 squared microns, there were less than 10% of objects counted (Fig 3A). In the simulations with 3-color nuclear labeling (no mixing), it was also clear that as the cell density increased, the number of objects counted decreased (Fig. 3D). However, the slope of the decline was not as steep as the 1-color nuclear labeling scenario (compare Fig. 3A with 3D). At a density of ~0.6 cells/100 squared microns, approximately 90% of the objects were counted and at the density of 1.6 cells/100 squared microns, there were still more than 50% of the objects counted (Fig. 3D).

The simulations with 3-color nuclear labeling (mixing) (Fig. 3B, C) had a much different shaped trend portraying the relationship in the number of counted objects versus increasing cell density. For example, the density range of 0.6 cells/100 squared microns to 1.6 cells/100 squared microns, the number of objects counted did not drop below 75% (Fig. 3E). However, for cell densities greater than 1.6 cells/100 squared microns, the number of objects counted decreased rapidly (Fig 3E). At 1.8 cells/100 squared microns, the number of objects counted was 50% of the total cell number, which was similar to the 3-color no mixing strategy (Fig 3E). Thus, in a typical 3-color (mixing) labeled embryo, the computer model predicted the expected percentage of number of objects counted, as determined by the highest number of simulations (10,000 total simulations), as between 70-74% (Fig. 3F).

In order to determine the typical variation in tissue density in the neural tube (and other tissues at early stages of development) and provide a comparison with the model predictions, we measured tissue densities in fluorescently labeled (DAPI) transverse tissue sections (Fig. 3G, H). We found that tissue density in the neural tube varied from approximately 1.7-4 cells/100 squared microns in embryos from E1.5-E3.5 (E = embryonic day). In comparison to the neural tube, we found that tissue density in the limb bud was ~3 cells/100 squared microns (Fig. 3G, H). Thus, analysis of different tissue densities in the early chick embryo and comparison to 3-color nuclear labeling (mixing and no-mixing) computer model predictions suggested expected object counting accuracies could vary from a high of ~90% to less than 35%.

### Single cell tracking by spectral identity improved the accuracy of identifying the appropriate cell when cell trajectories crossed paths

**Figure 4:**
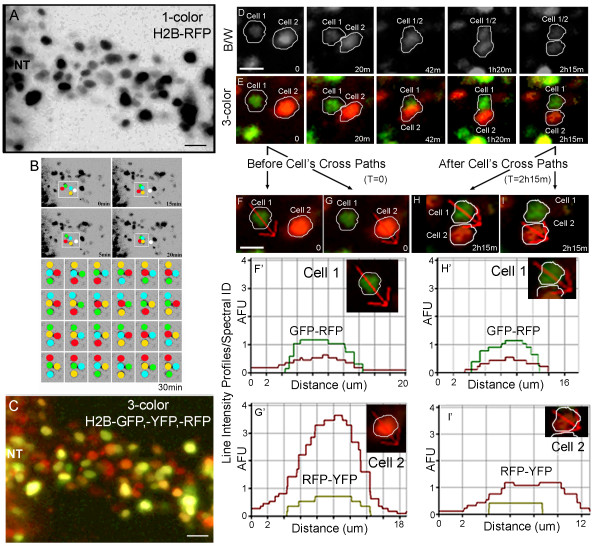
**Cell Tracking By Spectral Identification Resolves Cell Identity After Cell Trajectories Cross in Space and Time**. (A) A typical NC cell migratory stream at with cells labeled with H2B-RFP (grayscale: the images is inverted to more clearly visualize cells). (B) A closer look at a specific group of 4 migratory neural crest cells that cross pathways shows 24 (4-factorial, 4!) different combinations of possible cell identification after the cells move apart. (C) A typical 3-color nuclear labeled embryo reveals the NC cell migratory and highlights the advantage to visually distinguish individual cell nuclei. (D-E) More specifically, tracking two dynamic migratory NC cells that cross trajectories, the color information allows both visual inspection and spectral identity to distinguish the difference of the two cells when before and after the cells cross paths. Scale bars are 10 um. Throughout a typical time-lapse imaging session with a cell trajectory crossing, the individual cells are apart (0 min), approach each other (20 min), contact each other (42 min), then start to separate (1 h 20 min and 2 h 15 min). (F-G) Line intensity profiles were drawn manually (AIM) through the center of each cell. (F' & G') The line intensity profiles shown for Cell 1 and Cell 2 show the independent spectral identity of each cell. Cell 1 has an H2B-GFP and -RFP signature, while cell 2 has H2B-RFP and -YFP signature, creating an easily distinguishable pair of cells. (H & I) After the cells cross paths, line intensity profiles show the distinct spectral identities of each cell. The scale bars are 20 um (A&C) and (D-F) 10 um.

In this section, we addressed the second question of whether we could more efficiently track NC cell positions when cell trajectories crossed in space and time. Time-lapse imaging of 1-color H2B-RFP labeled embryos showed that when migratory NC cells moved close to each other, identification of distinct cell borders became difficult to determine, due to the limitations to resolve the distance between fluorescent pixels (Fig. 4A, D; see movie in Additional File 2). Automated cell tracking, based on cell shape and brightness, often had to guess on the appropriate cell, when cell trajectories crossed paths (Fig. 4B). That is, in a typical in vivo 1-color NC cell time-lapse imaging session, there may be hundreds of cell trajectory crossings and if multiple cells occupied nearly the same position in time (as judged by the computer), this led to n-factorial (n!) possibilities of subsequent cell tracking outcomes and significant tracking error potential (Fig. 4B). If these tracking errors were propagated, this led to significant differences in the accurate readout of cell positions (data not shown), a major problem for cell dynamics analysis.

We addressed this problem by using a 3-color H2B-FP NC cell labeling approach and 4D multispectral imaging (Fig. 1A, B, D, G; Fig. 4C). First, we found that when multiple H2B-FPs were introduced into the neural tube and NC cells, we could more accurately identify cell position based on visual inspection of time-lapse data (Fig. 4D, E; Additional File 3 and 4 for movies and Additional File 5). When NC cell trajectories crossed, we stepped backwards in the time-lapse sequence and used a line intensity profile to detect the spectral identification of each cell (Fig. 4F-I'). We then stepped forward in the time-lapse sequence and were able to accurately identify the same cells by matching the spectral properties (Fig. 4F-I'). When we introduced a cocktail of 3 H2B-FPs into NC cells, we found we could more accurately identify cell crossings since there were up to 7 combinations of FPs to distinguish in each NC cell (Fig. 4C).

### Multicolor labeled NC cells had a consistent short-term spectral identity as revealed during in vivo 4D multispectral confocal time-lapse imaging

To test whether a NC cell's spectral identity could be used as an additional parameter to track a cell's position in the embryo, it was necessary to monitor the spectral profile of a single NC cell during in vivo time-lapse sessions (Additional File 6). Specifically, we asked whether the relative ratio of multicolor fluorescent proteins within each migratory NC cell provided a stable spectral identification over the short-term (~5 hrs). To measure this quantity, we calculated the spectral profiles of many NC cells (m > 15) at each time point during 5-6 hr time-lapse imaging sessions (n = 5). We used spot detection of each cell to calculate the average pixel intensity values for each colored fluorescent protein (described in the Methods section). Interestingly, we found that the spectral identity of migratory NC cells remained consistent throughout short-term time-lapse imaging sessions, up to 5 hours (Additional File 5 &6). The exact peak or shape of the fluorescence intensities within a migratory NC cell changed throughout a time-lapse session, depending on the movement of the cell, but the hierarchical order of fluorescence intensities within the cell remained consistent (Additional File 5). For example, if a NC cell had bright H2B-GFP (green) and low H2B-mCherry (red) fluorescence, this feature remained consistent throughout the time-lapse imaging session (Additional File 5). We also found NC cell spectral identity remained consistent when a cell divided; the daughter cells had the same identity as the parent cell before the division (Additional File 6). Thus, this suggested that spectral identification could provide additional criteria for cell identification during tracking.

### Cell contact analysis was dramatically improved by combining 2-color cell membrane and 1-color nuclear labeling and 3D multispectral confocal imaging

**Figure 5:**
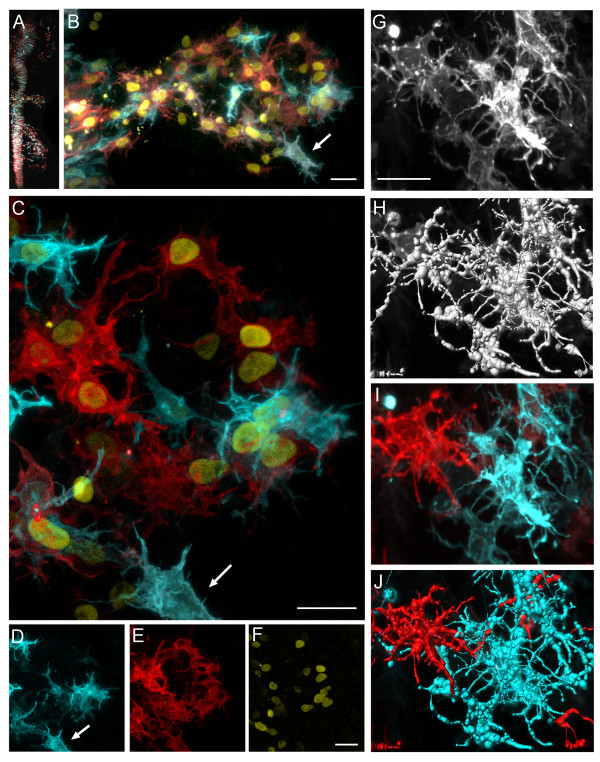
**Cell Contact Analysis Using 2-Color Membrane and 1-Color Nuclear Labeling in the Embryo Resolves Cell Interactions**. (A) A typical 6-8 somite embryo microinjected and electroporated with Gap 43-GFP, Gap43-YFP, and H2B-mCherry is shown. The GFP, YFP, and mCherry fluorescence signals have been pseudo-colored blue, red, and yellow, respectively, for better visual inspection. The distinct cranial NC cell migratory streams appear within the surrounding unlabeled tissue. (B-C) A higher magnification of the r4 NC cell migratory stream shows the intricate network of NC cellular processes. The arrow highlights a cell of interest at the migratory front. (D-F) The individual color channels from (C) show that not every cell is labeled with all three colors. Importantly, not all cells are labeled with both Gap43 constructs to distinguish individual cellular processes of neighboring cells. (G) A grey scale image highlights the advantage of using two different membrane labels. (H) Using semi-automated filament tracing software, NC cellular processes are rendered. However, it is difficult to distinguish between neighboring cells using both visual inspection and software. (I) Using the color information originally collected, a closer look at the same two cells (in G) reveals how the different colors highlight individual cellular processes and (J) segmentation shows the intricate network of neighboring cell contacts. All scale bars are 20 um.

One of the major challenges in analyzing NC cell dynamics has been the inability to resolve cellular processes between neighboring cells within a dense migratory stream. To begin to measure cell contact between neighboring NC cells more accurately, we labeled embryos with 2 distinct membrane localized fluorescent reporters (Gap43-FPs) and 1 nuclear localized fluorescent reporter (H2B-FP) (Fig. 1A, E, I). We first asked whether we could more efficiently detect cellular processes between NC cells by visual inspection, then utilize image processing software to trace cellular processes (Fig. 5A-F). We found that we could both visually and automatically trace individual filopodia from one NC cell to a neighboring NC cell (compare Fig. 5G, H with 5I, J). We were also able to measure the extent to which cellular processes interconnected between neighbors (compare Fig. 5G, H with 5I, J). Previously, it had been nearly impossible to distinguish which cellular process belonged to what cell, using a single color fluorescent reporter. Thus, this 3-color cell labeling strategy provided more accurate NC cell contact analysis while preserving the ability to track cells by nuclear label.

### Channel versus spectral unmixing decreased the time and effort of image acquisition and allowed for 2-photon dual excitation to resolve multicolor labeled neural crest cells

To test the difference in time and effort of image acquisition of 3-color labeled embryos, we compared the channel mode versus lambda scanning forms of spectral imaging (Fig. 1E; Table 1). Channel mode takes advantage of the ability to set prescribed limits on band pass ranges to collect emitted light during image acquisition. In 3-color labeled embryos (Gap43-GFP or Gap43-CFP, Gap43-YFP, H2B-mCherry), we collected 3D multispectral confocal z-stacks using either channel mode (3-track: 488/514/561 nm excitation) or lambda scanning (488/561 nm excitation; 494-623 nm, 10.7 nm step size). We compared the size of acquired data sets, time of image acquisition, and time to open and save 3D data sets (Table 1). We developed the following conclusions. First, lambda scanning was faster to acquire images. In a typical scenario (42 slices, 15 um z-stack with 0.36 um spacing; 1024 × 1024 pixels, scan speed = 7 (1.6 us pixel dwell time)), we found the lambda scanning to be 2 times faster (~11 min versus ~22 min) than channel mode (Table 1). However, the data set sizes for lambda scanning were over 5 times larger (880 MB versus 160 MB; Table 1). This significantly increased the time to save acquired images (~10 min versus 1 min; Table 1). Also, lambda scanning required an additional 5 min step to linearly un-mix images, however, in practice, temporal differences between scanning modes may be decreased by unmixing during acquisition. Thus, although lambda scanning was faster to acquire images of multicolor labeled embryos, channel mode produced data sets that were smaller and faster to open and manipulate in post-processing applications.

**Table 1:** Optical Parameters for Resolving 3-Color Labeled Cells in an Embryo (A Comparison of Channel vs Lambda Scanning and 2P Microscopy)

Combination	Scan Type			Other Information					
**3-Color**	**Channel Mode** **(3 channel)**	**Lambda Mode** **(Spectral Unmixing)**	**+ = good** **++ + = best**	**Single Photon (nm) Excitation** **Profiles**	**2P**	**2P Comments**	**1P Comments**		

Gap-43 GFP & Gap-43 YFP & H2B-mCherry	+ separation	++ separation^†^		3 track 488/514/561 2 track 488 + 561/514 1P Lambda 488/561	Yes	Overlap of GFP/YFP spectra in range 890-990 nm. Difficult to avoid dual excitation.	Reducing tracks to 2 saves time. May use 488 nm GFP/YFP or multiple laser combinations.		

Gap-43 CFP & Gap-43 YFP & H2B-mCherry	+++ separation^‡^	+++ separation^†^		3 track 405 or 458/514/561 2 track 405 or 458/561 + 514 1P Lambda 3 laser	Yes	Excitations to note: 800 nm : minimum YFP ex. 865 nm: max. CFP ex. 950 nm : max. YFP ex. 900 nm: max. dual ex.	May require 3 lasers. Available 1P excitation wavelengths may not be ideal (440 nm) for CFP.		

General Data Properties				Example Stack Comparison					

Property	Channel*	Lambda	Linear Unmixed	Frame: 1024 × 1024 × 15 μm (42 slice) Interval: 0.36 μm Avg: 4, Speed 7 (dwell 1.6 μs)					

Dataset Sizes (GB)	0.1 to 1	1 to 7	1/8^th ^stack size		Size	Scan Time	Save Time	Unmix Time	Unmix Size

Time to Image (Min./GB)	10* (1-10 min.)	8 (8-56 min.)	5 t_*unmix*_ Proportional to dataset size	Channel 8-bit 2 track 45s/slice	160 MB	22 min	<< 1 min	Not Applicable	Not Applicable

Time to Save (min.)	≤ 5	≈ t_*acquisition*_ (8-56 min.)	<< 1	Lambda 12-bit 15s/slice	880 MB	11 min	11 min	4:30 min	110 MB
						
Time to Open (Min./GB)	<< 1	1.5	<< 1						

Linear unmixing requires extra processing time.				Linear unmixing time advantages diminished at higher data set sizes					
* Channel data for three-track scanning. ‡ Rating based largely on time considerations. † Ratings roughly equivalent, however, CFP/YFP is more widely separated than GFP/YFP, and should generally perform better in unmixing.									

Computer properties	Windows Vista. Intel Dual Core Xeon x5260 3.33 MHz, 32-bit. 3 GB RAM. Data (D:) drive capacity 500 GB. Graphics ATI FireGL V5600 512 MB memory.								

Objective	LD C-Apochromat 40×/1.1 W Korr								

Lambda scan:	Depth (μm) 20 - 45 μm. Frame 1024 × 1024. 12-bit. Frame Avg. 4. Speed 7. Lasers 3% 488, 2.4% 561. Parameter ranges: Frame size: 512-1024, 8-16 bit. Frame avg: 2-8. Scan speed: 7-9.								

High-resolution imaging of whole mount chick embryos is possible on the upright 710. The manual nosepiece was configured for two objectives: a high N.A. immersion 40× and a 10×/0.45 or a 20×/0.8 dry objective. High-resolution images were taken in fluorescence using the 40× at variable zoom (1-3×) depending upon the region of interest. Overview images at 5-20× were taken in fluorescence and transmitted light, and were captured in either channel or lambda scanning. Scans were frame averaged at 4, scan speed of 7 (pixel dwell time: 1.6 us). The 40× slice dimension was typically 0.36 um. Laser power was kept below 5% owing to the sample brightness and detector sensitivity, particularly in lambda mode, where pixel overexposure must be prevented.

## Discussion

We used the chick neural crest (NC) as a model to study novel multicolor cell labeling and multispectral imaging strategies. We addressed two specific questions to more accurately identify and track in vivo NC cell positions and cell contact dynamics and discovered 5 main results. First, a comparison of nuclear targeted versus traditional (lipophilic dye or membrane GFP) cell labeling showed more accurate cell identification with nuclear labeling, but there was no significant difference in 1-color versus 3-color labeled tissues. Second, 3-color nuclear labeling significantly improved the ability to, both visually and with software, identify and track in vivo NC cell movements using spectral properties. Third, a theoretical model of object counting provided a useful tool to predict the expected accuracy of object counting in 1-color versus 3-color labeled tissue of increasing density. Fourth, analysis of cell contact dynamics was improved by the use of a 2-color membrane and 1-color nuclear labeling strategy. Fifth, the channel versus spectral unmixing method dramatically reduced the time and effort of image acquisition and manipulation of 3D multispectral confocal z-stacks.

It was not surprising that DiI cell labeling led to cell identification inaccuracies, since lipophilic dyes tend to become particled in the cell and not uniquely identify its shape (Fig. 2A, B, I). We anticipate that using quantum dots or fluorescent glass beads to label cells would in a similar manner not uniquely define an individual cell, although these fluorescent reporters may be brighter and resistant to photobleaching [17] and have other purposes. We were also not surprised that Gap43-GFP membrane labeling did not accurately identify individual NC cells, since it is typically difficult to visually detect distinct cell borders in vivo in dense tissues of the embryo (Fig. 2C, D, I). We expected a more significant difference in cell identification between 1-color and 3-color nuclear labeled embryos than the data revealed (Fig. 2I). This may have in part been due to the complications associated with segmentation of multi-color labeled cells. Future updates to image analysis software may take advantage of using 24 bit information where a 24-bit vector could be created from each pixel value (8-bits per RGB color). A monochrome image would then be created from this and the image segmented using a clustering method. In our current scenario, we introduced a cocktail of fluorescent plasmids by microinjection and electroporation in the early chick embryo (Fig. 1A, B). Since electroporation delivery has been shown effective in several embryo model systems, this should represent a relative easy means to generate multicolor nuclear labeled embryos. The multicolor cell labeling approach may further be simplified by the generation of 2A vectors that allow expression of multiple proteins from the same promoter.

Cell tracking by spectral identification offered additional criteria to more accurately determine a unique cell identity. In 3-color nuclear labeled embryos, we found that NC cells displayed different ratios of fluorescent reporters (Fig. 4). When NC cells occupied nearly the same position in space and time, as resolved by the light microscope, we were able to accurately identify cells as they moved apart, using their spectral profile (Fig. 4E-I'). This technique worked well since the use of multicolored fluorescent plasmids, introduced into cells in the chick embryos by electroporation, allowed up to 7 unique color combinations. Secondly, we found that multicolor labeled NC cells displayed a consistent ratio of fluorescent reporter intensities when followed in time-lapse sessions lasting at least 5 hours (Additional File 5 &6). In practice, we stepped backwards/forwards in a time-lapse sequence to compare the spectral identities of cells that crossed paths to acquire/re-acquire each cell (Fig. 4 and Additional File 6). We suggest that this type of method may be automated within existing cell tracking software. Thus, our results demonstrate that cell tracking using spectral information will offer additional criteria for cell and developmental biologists to uniquely identify a migratory cell in vivo and should become part of automated tracking software.

The computational model provided a predictive tool to determine whether using a 3-color nuclear labeling strategy would be worth the time and effort to produce a significantly different object counting accuracy (Fig. 3). The determination of time to generate a multicolor labeled embryo model may not be a consideration for embryo model systems in which the number of electroporated fluorescent plasmids can be interchanged rapidly. However, typically the generation of a transgenic embryo (e.g. mouse, Drosophila) takes significant time and effort. Second, depending on the tissue of interest within the embryo, there may be variation of cell densities over time (for example, shown in Fig. 3). Thus, the information predicted by the model would be helpful to have to weigh the decision whether or not to make the multicolor labeled embryo model.

Cell contact dynamics benefited from a 2-color cell membrane and 1-color nuclear cell labeling strategy. We found that cellular processes, such as filopodia could uniquely be distinguished on neighboring, different colored NC cells both by visual inspection and in semi-automated image segmentation (filament tracing) using separate color channel information (Fig. 5). We anticipate that this cell labeling strategy will now allow us to accurately calculate critical measurements of NC cell contact dynamics, including the number of neighboring cell contacts, persistence of contacts and whether cell contact leads to changes in cell morphology or direction. Cell labeling strategies of this type may help to resolve cell contact dynamics in other migratory cell populations and in dense tissue. To some extent, these types of cell labeling strategies have been applied to analyze axon genesis and dynamics [10,18] and structures such as dendrites identified with novel filament tracing software [18]. We found that by combining cell membrane and nuclear localized fluorescent reporters, we were able to measure cell contact dynamics and maintain an ability to track cell positions.

Channel mode versus lambda scanning forms of spectral imaging revealed the trade-offs between each form of image collection (Fig. 1E; Table 1). Although lambda scanning was faster, it was more efficient to save and manipulate images collected with channel mode (Table 1). We anticipate that the comparisons shown here should serve as a starting framework for the decision of which mode to employ and may also depend on the embryo model system and photobleaching and photo toxicity measurements.

## Conclusions

In summary, we have presented an investigation of novel multicolor cell labeling and 3D/4D multispectral confocal and 2-photon imaging strategies in the embryo and show improvements in cell tracking and cell contact analyses using spectral information. We chose to test these strategies in a migratory cell type, the neural crest, and chick embryo model, however the concepts are widely applicable to other model systems. Our results demonstrate that multicolor cell labeling and multispectral imaging provide a unique cellular fingerprint. This cellular fingerprint, obtained by spectral identification, over the short-term more accurately determines a cell's position and contacts with other cells and tissue within the embryo. Thus, multicolor cell labeling and multispectral imaging offer a powerful spectral toolbox in the field of intravital imaging and a means to break down and interpret the complex choreography of cell movements during embryogenesis.

## Methods

### Embryo Development

Chick eggs were received from Sean Pryor Farms (Oeosho, MO) and incubated at 37°C until maturation to the 8-9 somite stage [19]. The eggs were lightly sprayed with 70% Ethanol and 3 ml of albumen was removed from the large end of the egg using a 5 mL syringe (309603, Becton Dickinson, Franklin Lakes, NJ, USA) and an 18-gauge needle (305196, Becton Dickinson). Embryos were staged by cutting a hole in the eggshell and injecting India ink (PLK 51822A143; Pelikan Fount; Houston, TX; 1:10 in Howard Ringer's solution) underneath the embryo using a 1 mL syringe (309628, Becton Dickinson) and a 25-gauge needle (305112, Becton Dickinson) to provide contrast under a stereomicroscope (MZ9.5; Leica Microsystems, Bannockburn, IL).

### Fluorescent Labeling of Neural Crest Cells

Fluorescent DNA plasmids were microinjected directly into the lumen of the chick neural tube of HH Stage 8-9 embryos at the axial level of rhombomere 3. The plasmids used included nuclear-localizing H2B-GFP, -YFP, -RFP, -CFP and -mCherry as well as membrane-localizing Gap43-GFP, -CFP and -YFP (kind gifts from Rusty Lansford and Mark Parrish). All plasmids were used at an initial concentration of 5 ug/ul. For simultaneous multicolor labeling of NC cells, a cocktail of up to 3 different fluorescent plasmids was mixed, at equal ratios, within a single filamented glass needle (BF100-50-10; Sutter Instruments, Novato, CA). A micromanipulator (M3301R; World Precision Instruments; Sarasota, Florida) and microinjector (Picospritzer III, Parker Hannifin Corporation; Cleveland, OH) were used for targeted delivery of the fluorescent reporters into the neural tube. Electroporation (Electro Square Porator ECM 830; 45-0002; BTX Harvard Apparatus; Holliston, MA), was performed with proper placement of electrodes (711000; A-M Systems; Carlsborg, WA) on either side of the neural tube allowing for fluorescent plasmids to be directed into cells on the right-hand side of the neural tube. Pulses of 20 volts of 45 ms were dispensed through the electrodes 5 times, with 1 second between each pulse. In some experiments, DiI was microinjected directly into the lumen of the chick neural tube of HH stage 8-9 embryos at the axial level of rhombomere 3. The lipophilic dye, DiI (C-7000; Invitrogen, Molecular Probes; San Diego, CA), was mixed with ethanol and 3 M sucrose (50 ug:10 ul: 90 ul) to produce a 500 ug/ml solution. After fluorescent cell labeling, embryos were re-incubated at 37°C for 6-24 hrs (6 hrs for time-lapse imaging and 24 hrs for static imaging) and then surveyed for brightness and distribution of fluorescence throughout the hindbrain using a fluorescence dissection microscope (Leica MZFL III).

### Tissue Preparation

Transverse sections of chick embryos were cut with a vibratome (VT1000S; Leica Microsystems; Bannockburn, IL) into 60 um slices from the r2 to r7 axial levels. Transverse tissue sections were cut for visualization of the neural tube and migratory neural crest cells in surrounding tissue. Tissue sections were laid sequentially in rows on glass micro slides (48312-024; VWR; West Chester, PA) in a region surrounded by silicone grease (79810-99; Dow Corning; Midland, MI) and sealed with a glass coverslip (48366-089; VWR; West Chester, PA). For tissue density approximations, we counted the number of nuclear-labeled cells in a 100-micron squared region of the tissue. An alternative and perhaps more informative measurement would be to calculate the separation between nuclei, approximated by finding the average distance between nuclear centers minus the average nuclear diameter.

### 3D and 4D Multispectral Confocal Imaging

Each tissue section described above was imaged with either channel or lambda scanning using single photon excitation on an LSM 510 META (Carl Zeiss; Thornwood, NY) with either a 20× (NA = 0.8) or 10× (NA = 0.45) Plan-Apochromat objective (Zeiss). 3D z-stacks were acquired, using a frame size of 512 × 512 or 1024 × 1024 with 12-bits. A scan speed of 2.56 usec/pixel, a pinhole of approximately 1 Airy unit, and line averaging using the mean of 2-4 scans was used for static imaging. A scan speed of 3.20 usec/pixel, a pinhole of approximately 5 Airy units, and line averaging using the mean of 2 scans was used for live cell imaging. Our intravital cellular resolution was limited due to the working distance between the objective and our whole embryo explant culture, and fewer numbers of z-sections collected in order to maintain embryo health with less phototoxicity. The 488 nm laser line was produced by a 30 mW argon laser with a 7% transmission percentage. A transmission percentage of 10% was used from a 15 mW diode-pumped solid-state laser to produce the 561 nm laser light. In each imaging scenario, the 405/488/561 notch filter was in place to reflect the 488 nm and/or 561 nm laser light in the direction of the sample.

DiI-labeled sections were imaged using lambda scanning on the META in a 10.7 nm step size for signal collection ranging from 571-657 nm. Each 3D z-stack was acquired with an approximate 0.90 um step size. All sections labeled with membrane GFP used a 488 nm laser at 10% in channel mode using a BP 505-550 nm filter. For tissue sections expressing only H2B-RFP, the signal was divided into 8 channels (lambda scanning (META) detected signal ranging from 571-657 nms using a step size of 10.7 nms with the z-stack interval of 0.90 um). For tissue sections expressing 3 fluorescent proteins, signal was collected on the META for wavelengths 494-623 nm using a 10.7 nm step size to produce a total of 12 channels with the z-stack interval of 0.81 um.

### Linear Unmixing

Each set of static lambda 3D confocal z-stacks was linearly unmixed with AIM software (Zeiss) using reference spectra calculated from HEK 293 cells (CRL-1573; ATCC; Manassas, VA) fluorescently labeled with either DiI, H2B- GFP, -YFP, -CFP, -mCherry or -RFP to subtract out autofluorescence from the chick embryo tissue. The contribution of each spectrum was applied to each pixel within an image using the AIM software. Briefly, unmixed images were opened with each channel represented in its own window to perform a maximum intensity projection that resulted in a single image formed from the z-stack. For each channel, a maximum background value was calculated from a rectangle drawn in the background (no fluorescent signal present). A value of 125% of this maximum value was used as the threshold for that channel and was applied to the original z stack (25% was added to make sure there were background pixels; zero was used as a threshold to count cells such that every pixel with a grey value above zero would be considered a cell).

### Cell Identification (Automated and Manual)

Image J (NIH) eliminated all background such that the spots threshold was set to zero in order to assume any signal was produced from fluorescent label. Background subtracted images were loaded into Imaris (Bitplane AG; Saint Paul, MN) and analyzed to determine localized regions of relatively higher intensity signal (objects). For the automatic cell counts, each object identified in Imaris was considered a cell (estimated diameter per cell was 2 um for DiI, 5 um for H2B label, and 10 um for membrane GFP based upon conservative approximations calculated for cell diameter of each construct) and a total number of labeled cells in the image was calculated. Furthermore, Matlab (The MathWorks, Inc; Natick, MA) was used within the Imaris program to link DiI-labeled spots within 5 um due to the sparse and punctuate labeling of the dye (each group of DiI-linked spots was scored as a cell to determine the number of DiI labeled cells within an image). For the 3-color H2B nuclear labeling, each channel (red, green, yellow) was run through automatic spot detection, then the 3 channels were combined. Spots less than 2 um apart were linked (in Matlab) to account for the cells labeled with more than one color. For the manual cell counts the background subtracted images were loaded (into Imaris) and counted manually in 3D.

### Cell Tracking by Spectral Identity

For cell tracking, spectral profiles of individual cells were measured from the original 3-color H2B-labeled images using the AIM software. Profiles were measured from individual cells prior to cell trajectory crossing (visually identified), at the time point when neighboring cells could not be optically resolved as two distinct objects, and after cell separation. The spectral identities of the individual cells throughout time were exported and graphed according to time in Excel. The cells were also tracked in Imaris manually to show the 3-color labeling allowed proper identification of each cell.

### Channel versus Spectral Unmixing

Whole mount embryos were placed on glass micro slides (48312-024; VWR; West Chester, PA) in a region surrounded by silicone grease (79810-99; Dow Corning; Midland, MI) and sealed with a glass coverslip (48366-089;VWR; West Chester, PA). The embryos were imaged on an upright Zeiss LSM 710 (Carl Zeiss; Thornwood, NY) using a 5× (NA = 0.25) Fluar, 20× (NA = 1.0) W Plan-Apochromat, and 40× (NA = 1.1) W LD C-Apochromat. All images were taken at 1024 × 1024, 12 bit with frame averaging of 4-8, speed of 7 (pixel dwell time of 1.6 us), with a pinhole around 1 airy unit. Typically, the z-stack dimension was approximately 0.36 um per slice with a stack size ranging from 20-50 um. Laser power of the 405, 488, 514, 561, or 860 (two photon) were approximately 5% for each laser to ensure no over exposed pixels (especially in lambda scanning). In lambda mode, the 488/561 filter was used for GFP, YFP, and mCherry and unmixing was completed afterward from reference spectra obtained from embryos labeled individually with each color. If YFP and GFP were used in the same sample, lambda scanning was essential to ensure that the colors could be linearly unmixed since the emission spectra overlap. If CFP was used, the two photon or 405 nm lasers worked equally well. In channel mode for CFP, YFP, and mCherry, two channels were used with the 458/561, 405 filter for the red and blue channel, and the yellow channel used the 458/514 filter.

## Authors' contributions

RM, JMT, and MS performed all the embryo labeling, sample preparation, multispectral imaging, and image analysis in the DiI, GFP, and multi-color H2B-labeling experiments and cell tracking. CHJC and JMT performed all the embryo labeling, sample preparation, multispectral imaging, and image analysis in the multi-color cell membrane labeling experiments. RA designed and carried out the computational model simulations. RL provided many of the fluorescent protein constructs and participated in discussions of the experimental design strategy. PMK designed the research, analyzed data and wrote the paper. All authors read and approved the final manuscript.

## Supplementary Material

Additional file 1 **Methods for Computer Model Simulation**. The computer model simulation was used to predict counting accuracy for increasing densities. All computer simulations were written in Java code in ImageJ. Click here for file

Additional file 2 **Time-lapse analysis reveals that single-colored individual neural crest cells are difficult to follow when cell trajectories cross**. Cranial NC cells migrate from the neural tube (right-hand side) towards a peripheral target (left-hand side). During the migration process, NC cells may cross trajectories (at the same place and time), making it difficult to precisely follow both cell trajectories when the neighboring cells move apart. During this typical time-lapse sequence (n = 8), two NC cells cross paths, then move apart, making it difficult to determine which cell is which by both visual inspection and an automated cell tracking algorithm. The time between frames is 5 min and the total time is approximately 2 hrs. Each cell nucleus (H2B-RFP labeled) is approximately 10 um. Click here for file

Additional file 3 **Time-lapse analysis reveals that multicolor-labeled neural crest cells are easier to follow when cell trajectories cross**. The same movie as Movie 1, but with all of the recorded channels visible to reveal multicolor-labeled NC cells. During the migration process, the NC cells that cross trajectories are more easily identified after the cells move apart. The time between frames is 5 min and the total time is approximately 2 hrs. Each cell nucleus (H2B-GFP and H2B-RFP labeled) is approximately 10 um. Click here for file

Additional file 4 **Time-lapse analysis reveals that multicolor-labeled cell movements are traceable within the dense neural tube**. Cells within the neural tube, multicolor-labeled with either H2B-GFP or H2B-RFP or both, move between the apical-luminal (left-hand side) and basal (right-hand side) sides of the neural tube. During this typical time-lapse sequence (n = 8), cells exchange positions with neighboring cells and may divide. The time between frames is 5 min and the total time is approximately 5 hrs. Each cell nucleus is approximately 10 um. Click here for file

Additional file 5 **Multicolor-Labeled Neural Tube Cells are Tracked More Accurately in Dense Tissue and Show Consistent Spectral Identity Following Cell Division**. (A-C) Cells within one-side of the neural tube were labeled with a cocktail of H2B-GFP and H2B-RFP in the hindbrain between rhombomere 3 (r3)-r5. A confocal section through the neural tube is shown and the apical (a; luminal side), and basal (b) side are shown. The subregion of the neural tube is denoted by the asterisk. (D-L') In a typical time-lapse session, multicolor-labeled cells within the neural tube, with cell density of 2-3 cells per 100 micron-squared region, are more readily identified by spectral identification and are visually traced over time. (D) In this example, cell 1 (near apical side of the neural tube) has just divided (progeny = cell 1') at 6 h (h = hours) and (E) cells 1-4 are aligned from the apical to basal wall. (F) Following these cells backward in the time-lapse, it is evident that cells 1 and 4 switch positions between the apical and basal sides and (G) cells 1-4 start in a cluster, rather than in a line. (H-L') By tracing the spectral profile through cells 1 and 4, it is clear that there is a unique profile for each cell that assists in cell identification as the cells are apart (H-I'), move in close proximity (J-J'), then move apart (K-L'). The time-lapse lasts for 6 h with an interval of 6.5 minutes between frames. (M-U) Multicolor-labeled NC cells retain their spectral identification after cell division. (M-R) Multicolor-labeled NC cells emerging from r4 divide during migration. (S) Calculation of the spectral profile of a NC cell during division show the fluorescence intensities of the 2-color labeled NC cell, that is (T, U) consistently retained in both daughter cells. That is, the red fluorescence remains higher than the green signal throughout the time-lapse and the daughter cells display the same red higher than green intensity profile, therefore showing the intensity profile of the daughter cells are the same as the parent. The scale bars are (A) 100 um, (B, C) 50 um, (D-G) 20 um, (H-L) 20 um, (M, N) 40 um and (O-R) 5 um. Click here for file

Additional file 6 **NC Cell Spectral Identity Remains Consistent in Short Term (~5 hr) Time-Lapse Imaging **(A) A typical NC cell migratory stream labeled with H2B-mCherry, -YFP (shown in green), and CFP is shown in images from a 4 hr time-lapse imaging session. A maximum intensity projection of the z-stack is shown every 50 minutes. Specifically, a migratory NC cell (circled and pointed to by an arrow) is tracked by spot detection throughout the time-lapse. The spot detected was used to determine the average intensity of each color (red, green, and blue) of the cell. (B) The average intensity of each color was combined and a percentage of this total was calculated for each of the 3 colors throughout the time-lapse. Although each fluorescence intensity had some variability, the ratios remained approximately constant. Click here for file

## References

[B1] Kulesa PM Bailey CM Kasemeier-Kulesa JC McLennan R Cranial neural crest migration: New rules for an old road Dev Biol 2010 344 2 543 54 10.1016/j.ydbio.2010.04.010 20399765 PMC2914193

[B2] Gammill LS Roffers-Agarwal J Division of labor during trunk neural crest development Dev Biol 2010 344 2 555 65 10.1016/j.ydbio.2010.04.009 20399766 PMC2914176

[B3] Shaner NC Steinbach PA Tsien RY A guide to choosing fluorescent proteins Nat Methods 2005 2 12 905 9 10.1038/nmeth819 16299475

[B4] Lansford R Bearman G Fraser SE Resolution of multiple green fluorescent protein color variants and dyes using two-photon microscopy and imaging spectroscopy J of Biomed Optics 2001 6 3 311 318 10.1117/1.1383780 11516321

[B5] Dickinson ME Bearman G Tilie S Lansford R Fraser SE Multi-spectral imaging and linear unmixing add a whole new dimension to laser scanning fluorescence microscopy Biotechniques 2001 31 6 1272 1274-6, 1278 11768655 10.2144/01316bt01

[B6] Finley KR Davidson AE Ekker SC Three-color imaging using fluorescent proteins in living zebrafish embryos BioTechniques 2001 31 66 72 11464522 10.2144/01311st02

[B7] Hutter H Five-color in vivo imaging of neurons in Caenorhabditis elegans J Microsc 2004 215 213 218 10.1111/j.0022-2720.2004.01367.x 15315508

[B8] Paddock SW Langeland JA Devries PJ Carroll SB Three-color immunofluorescence imaging of Drosophila embryos by laser scanning confocal microscopy BioTechniques 1993 14 42 48 8424875

[B9] Hadjantonakis AK Macmaster S Nagy A Embryonic stem cells and mice expressing different GFP variants for multiple non-invasive reporter usage within a single animal BMC-Biotechnology 2002 2 11 10.1186/1472-6750-2-11 12079497 PMC116589

[B10] Livet J Weissman TA Kang H Draft RW Lu J Bennis RA Sanes JR Lichtman JW Transgenic strategies for combinatorial expression of fluorescent proteins in the nervous system Nature 2007 450 52 62 10.1038/nature06293 17972876

[B11] Teddy JM Lansford R Kulesa PM Four Color, 4-D time-lapse confocal imaging of chick embryos BioTechniques 2005 39 5 703 709 10.2144/000112017 16312219

[B12] Hadjantonakis AK Papaioannou V Dynamic in vivo imaging and cell tracking using a histone fluorescent protein fusion in mice BMC- Biotechnology 2004 4 33 1 1 15619330 10.1186/1472-6750-4-33 PMC544401

[B13] Bao G Murray JI Boyle T Ooi SL Sandel MJ Waterston RH Automated cell lineage tracing in Caenorhabditis elegans PNAS 2006 103 8 2707 12 10.1073/pnas.0511111103 16477039 PMC1413828

[B14] England SJ Adams RJ Building a dynamic fate map BioTechniques 2007 43 20 24 10.2144/000112510 17933098

[B15] Kulesa PM Fraser SE Neural crest cell dynamics revealed by time-lapse video microscopy of whole embryo chick explant cultures Dev Biol 1998 204 2 327 44 10.1006/dbio.1998.9082 9882474

[B16] Kulesa PM Fraser SE In ovo time-lapse analysis of chick hindbrain neural crest cell migration shows cell interactions during migration to the branchial arches Dev 2000 127 1161 1172 10.1242/dev.127.6.1161 10683170

[B17] Reiger S Kulkarni RP Darcy D Fraser SE Koster RW Quantum dots are powerful multipurpose vital labeling agents in zebrafish embryos Dev Dyn 2005 234 3 670 681 10.1002/dvdy.20524 16110511

[B18] Brown CE Ping L Boyd JD Delaney KR Murphy TH Extensive turnover of dendritic spines and vascular remodeling in cortical tissues recovering from stroke J Neurosci 2007 27 15 4101 4109 10.1523/JNEUROSCI.4295-06.2007 17428988 PMC6672555

[B19] Hamburger V Hamilton HL A series of normal stages in the development of the chick embryo J Morph 1951 88 49 92 10.1002/jmor.1050880104 24539719

