# Supplementary material for: Multispectral fingerprinting for improved in vivo cell dynamics analysis
Source: BMC Dev Biol. 2010 Sep 24;10:101. doi: 10.1186/1471-213X-10-101 (PMC2954993; doi:10.1186/1471-213X-10-101)
Supplement: Additional file 1 — Methods for Computer Model Simulation. The computer model simulation was used to predict counting accuracy for increasing densities. All computer simulations were written in Java code in ImageJ. [file 1471-213X-10-101-S1.DOC]

**METHODS:**

**Computer Model Simulations**

The computer simulations were written in java code in ImageJ with the following three assumptions. Assumption 1: By surveying the most densely packed region in the neural tube in several images, we found there were approximately 100 cells, each with an average size of 48 square microns, within a 5450 square micron area. Using a 20X objective, the scaling factor was 1.14 pixels per um, so the simulation was performed in a 7,000 square pixel area and each cell had a size of 65 square pixels. Assumption 2: All cells were placed at random within the frame, with the single restriction that the center of a cell could not overlap any part of another cell. This restriction simulated the nuclear labeling and the random label distribution due to electroporation. Assumption 3: To simulate the multicolor efficiency color labeling, each of the three colors was assigned a probability of labeling a cell. A random number generator was used to decide whether or not a cell was labeled based on the assigned color probability.

The overall flow for the simulation included the following: the numbers of red, green, and blue cells were selected, a random pixel was selected to be the new cell center, and then a random number generator decided whether or not a cell was labeled based on the assigned color probability. The number of cells in a simulation for each group ranged from 30<N<300. The center pixel was black, that is, no part of another cell could occupy this space. Next, a random number was tested against probability to see if a red cell should be drawn or the program should test another number. This was repeated for all three colors until the loop had created the number of cells requested for the simulation. The script was repeated for many different color combinations illustrated by the following table (below).

Each color possibility began with 10 cells, and incremented to 100 by 10. This yielded 1,000 color combinations. Non-unique permutations were grouped together during data analysis. Moreover, each color combination was repeated 10 times to give statistical merit to every color combination, producing a total of 10,000 simulations. There were three different cases of the simulation performed. A one color simulation, a three color no mix simulation and a three color mix simulation. The three color no mix meant the cells could only have one color in the cell body, but there are three colors that could be used for the whole population of cells. The three color mix was defined that a cell could have as many as three colors in the cell body. When Axiovision (Zeiss) performed object counting, it made no distinction as to the shape or size of objects counted. A counted object could be a single cell or a cluster of cells that could not be spectrally separated (Fig. 3C). All percentages in the Results and Fig. 3 should be taken to mean **number of objects counted** divided by **number of total cells.**

| **Number of Cells** | | |
| --- | --- | --- |
| **Red** | **Green** | **Blue** |
| 10 | 10 | 10 |
| 10 | 10 | 20 |
| 10 | 10 | 30 |
| … | … | … |
| 10 | 20 | 10 |
| 10 | 20 | 20 |
| 10 | 20 | 30 |
| … | … | … |
| 20 | 10 | 10 |
| 20 | 10 | 20 |
| 20 | 10 | 30 |
| … | … | … |
| 100 | 100 | 100 |
