# Supplementary figures and images for: Multispectral fingerprinting for improved in vivo cell dynamics analysis
Source: BMC Dev Biol. 2010 Sep 24;10:101. doi: 10.1186/1471-213X-10-101 (PMC2954993; doi:10.1186/1471-213X-10-101)

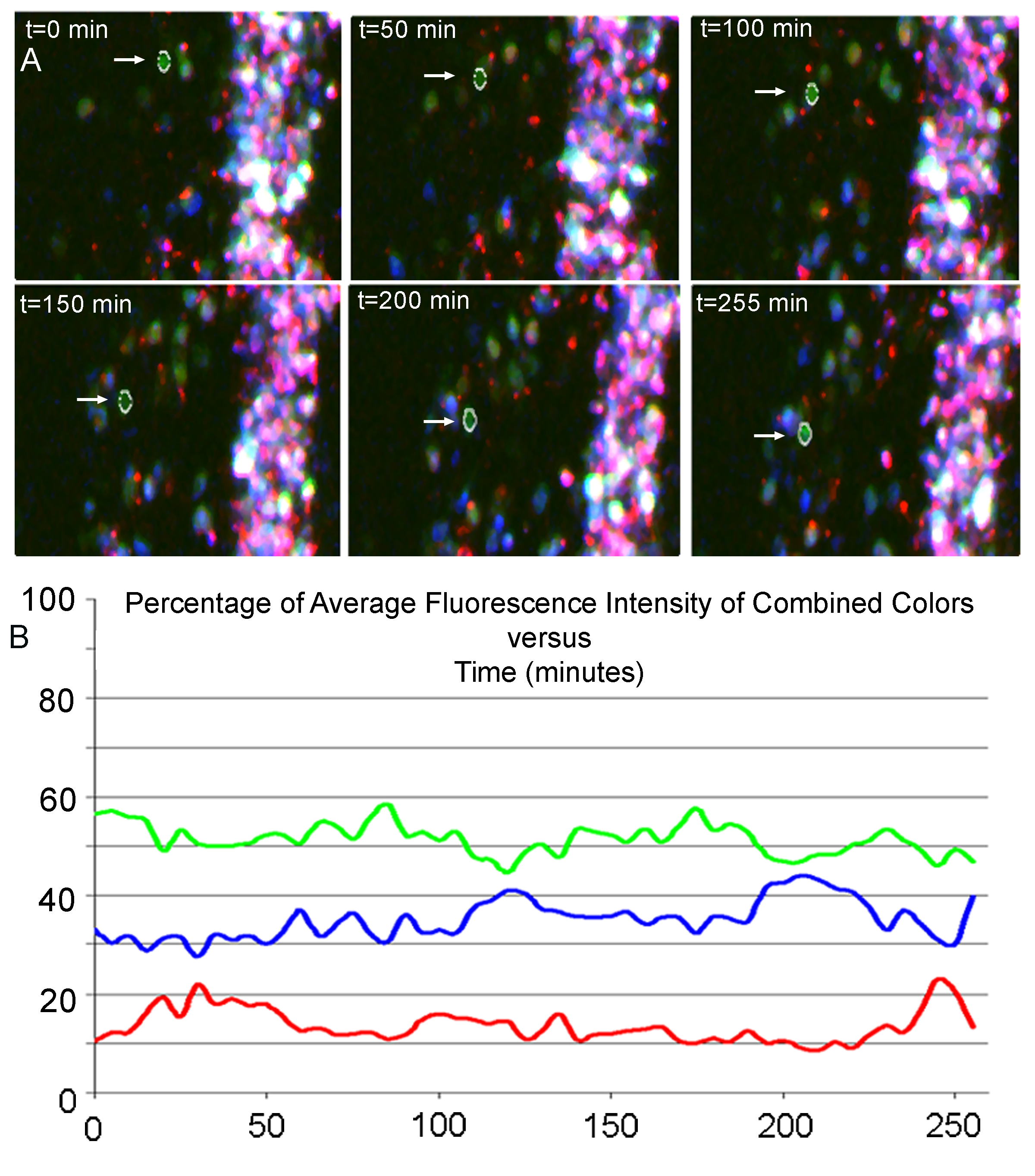

Supplement: Additional file 5 — Multicolor-Labeled Neural Tube Cells are Tracked More Accurately in Dense Tissue and Show Consistent Spectral Identity Following Cell Division. (A-C) Cells within one-side of the neural tube were labeled with a cocktail of H2B-GFP and H2B-RFP in the hindbrain between rhombomere 3 (r3)-r5. A confocal section through the neural tube is shown and the apical (a; luminal side), and basal (b) side are shown. The subregion of the neural tube is denoted by the asterisk. (D-L') In a typical time-lapse session, multicolor-labeled cells within the neural tube, with cell density of 2-3 cells per 100 micron-squared region, are more readily identified by spectral identification and are visually traced over time. (D) In this example, cell 1 (near apical side of the neural tube) has just divided (progeny = cell 1') at 6 h (h = hours) and (E) cells 1-4 are aligned from the apical to basal wall. (F) Following these cells backward in the time-lapse, it is evident that cells 1 and 4 switch positions between the apical and basal sides and (G) cells 1-4 start in a cluster, rather than in a line. (H-L') By tracing the spectral profile through cells 1 and 4, it is clear that there is a unique profile for each cell that assists in cell identification as the cells are apart (H-I'), move in close proximity (J-J'), then move apart (K-L'). The time-lapse lasts for 6 h with an interval of 6.5 minutes between frames. (M-U) Multicolor-labeled NC cells retain their spectral identification after cell division. (M-R) Multicolor-labeled NC cells emerging from r4 divide during migration. (S) Calculation of the spectral profile of a NC cell during division show the fluorescence intensities of the 2-color labeled NC cell, that is (T, U) consistently retained in both daughter cells. That is, the red fluorescence remains higher than the green signal throughout the time-lapse and the daughter cells display the same red higher than green intensity profile, therefore showing the intensity profile of the daugh [file 1471-213X-10-101-S5.TIFF]

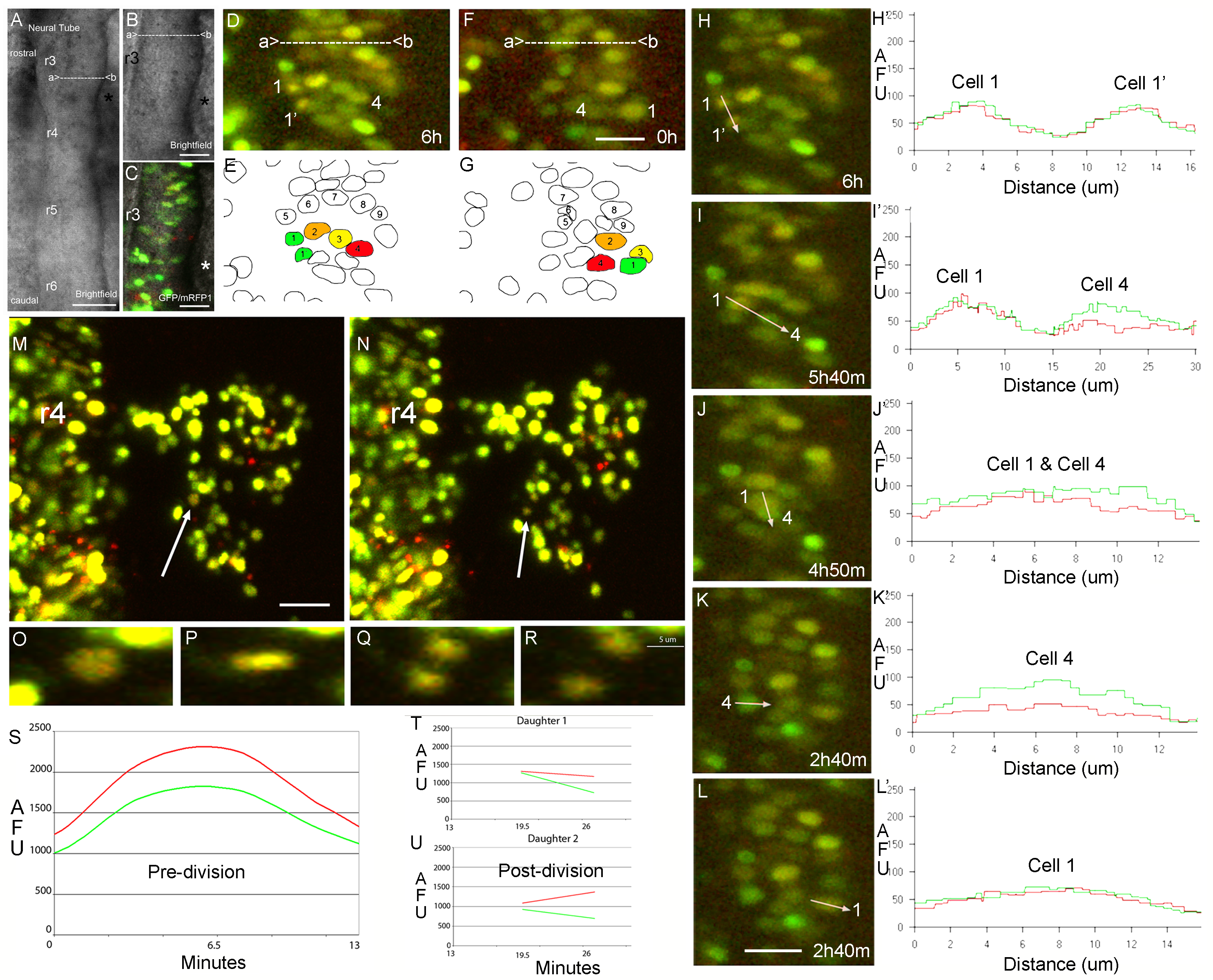

Supplement: Additional file 6 — NC Cell Spectral Identity Remains Consistent in Short Term (~5 hr) Time-Lapse Imaging (A) A typical NC cell migratory stream labeled with H2B-mCherry, -YFP (shown in green), and CFP is shown in images from a 4 hr time-lapse imaging session. A maximum intensity projection of the z-stack is shown every 50 minutes. Specifically, a migratory NC cell (circled and pointed to by an arrow) is tracked by spot detection throughout the time-lapse. The spot detected was used to determine the average intensity of each color (red, green, and blue) of the cell. (B) The average intensity of each color was combined and a percentage of this total was calculated for each of the 3 colors throughout the time-lapse. Although each fluorescence intensity had some variability, the ratios remained approximately constant. [file 1471-213X-10-101-S6.TIFF]
